# Exercise pressor reflex function following acute hemi-section of the spinal cord in cats

**DOI:** 10.3389/fphys.2013.00003

**Published:** 2013-02-07

**Authors:** Megan N. Murphy, Ronaldo M. Ichiyama, Gary A. Iwamoto, Jere H. Mitchell, Scott A. Smith

**Affiliations:** ^1^Department of Physical Therapy, University of Texas Southwestern Medical CenterDallas, TX, USA; ^2^Department of Applied Physiology and Wellness, Southern Methodist UniversityDallas, TX, USA; ^3^School of Biomedical Sciences, University of LeedsLeeds, UK; ^4^Department of Cell Biology, University of Texas Southwestern Medical CenterDallas, TX, USA; ^5^Department of Internal Medicine, University of Texas Southwestern Medical CenterDallas, TX, USA

**Keywords:** blood pressure, heart rate, muscle afferents, spinal cord injury, cardiovascular disease

## Abstract

Cardiovascular disease is a leading cause of morbidity and mortality in patients post spinal cord injury (SCI). The prescription of exercise as a therapeutic modality for disease prevention in this population is promising. It is logical to suggest that the sooner an exercise program can begin the more benefit the patient will receive from the therapy. However, the time point after injury at which the requisite circulatory responses needed to support exercise are viable remains largely unknown. The skeletal muscle exercise pressor reflex (EPR) significantly contributes to cardiovascular control during exercise in healthy individuals. Experiments in patients with a chronic lateral hemi-section of the spinal cord (Brown-Séquard syndrome) suggest that the EPR, although blunted, is operational when examined months to years post injury. However, whether this critically important reflex remains functional immediately after lateral SCI or, in contrast, experiences a period of reduced capacity due to spinal shock has not been established. This study was designed to assess EPR function after acute lateral transection of the spinal cord. The EPR was selectively activated in seven decerebrate cats via electrically stimulated static contraction of the triceps surae muscles of each hindlimb before and after lateral hemi-section of the T_13_–L_2_ region of the spinal cord. Compared to responses prior to injury, increases in mean arterial pressure (MAP) were significantly decreased when contracting the hindlimb either ipsilateral to the lesion (MAP = 17 ± 3 mmHg before and 9 ± 2 mmHg after) or contralateral to the lesion (MAP = 22 ± 5 mmHg before and 12 ± 4 mmHg after). The heart rate (HR) response to stimulation of the EPR was largely unaffected by induction of acute SCI. The findings suggest that the EPR maintains the ability to importantly contribute to cardiovascular regulation during exercise immediately following a Brown-Séquard-like injury.

## Introduction

Spinal cord injury (SCI) often leads to a reduction in physical activity and a loss of muscle mass as well as the generation of significant autonomic cardiovascular dysfunction (Hagen et al., 2012). In part as a result of these maladaptive responses to injury, the development of cardiovascular disease post SCI is a major concern and is now recognized as a leading cause of morbidity and mortality in the SCI patient (Myers et al., 2007). It has long been known that various forms of exercise can improve physical and cardiovascular fitness in individuals with SCI (Hicks et al., 2011; Phillips et al., 2011). Thus, the prescription of exercise as a therapeutic modality to prevent the development of cardiovascular disease after SCI is promising. It stands to reason that the earlier an exercise program can be undertaken in this patient group, the greater the impact the physical training will have as a preventative and health promoting measure. This being stated, the time point after injury at which the requisite cardiovascular adjustments needed to support exercise are operative remains relatively unknown. Therefore, understanding both the timing at which cardiovascular regulation is viable and the mechanisms underlying this control after SCI is critically important to the design and prescription of exercise in this patient population.

During exercise, two primary neural inputs are engaged that regulate the cardiovascular response to physical activity. The first, central command, is a feed-forward mechanism originating within the cerebral cortex that activates both descending motor neurons and cardiovascular control circuits in a parallel fashion (Goodwin et al., 1972). The second, the exercise pressor reflex (EPR), is a feed-back mechanism that likewise activates cardiovascular control centers when mechanically and chemically sensitive Group III and IV afferent fibers originating in skeletal muscle are stimulated during contraction (Kaufman et al., 1983; Mitchell et al., 1983; Hayes et al., 2005). Combined input from these two neural sources mediates the requisite circulatory adjustments needed to meet the metabolic demands of working skeletal muscle; primarily, sympathetically mediated increases in blood pressure and cardiac function (Smith et al., 2006; Murphy et al., 2011). Given the significance of both central command and the EPR to neural cardiovascular control, it is important to understand the contribution of each during exercise after SCI.

To this end, a previous study was performed in patients designed to determine the contribution of central command and the EPR to the cardiovascular response to static exercise post SCI (Winchester et al., 2000). The investigation utilized individuals with Brown-Séquard syndrome; a condition characterized by a unique lateral hemi-section of the spinal cord which leaves the ipsilateral side of the body below the lesion with reduced motor function and normal sensation while the contralateral side typically has normal motor function and decreased sensation to stimuli transduced by small diameter afferent fibers. In the study, the cardiovascular responses elicited by both voluntary and electrically stimulated contractions of the limbs led the investigators to conclude that central command and the EPR remained operative maintaining their ability, at least in part, to induce the circulatory adjustments required to perform physical activity. While the investigation provided significant insight into the mechanisms of neural cardiovascular control in Brown-Séquard patients, it did so in a patient population that was months to years post injury. Whether this control is adequate to support exercise immediately following this type of SCI remains unknown. The EPR could be particularly susceptible to dysfunction directly after lateral spinal hemi-section due to the development of spinal shock; a condition in which reflexes originating caudal to the site of the spinal cord lesion are transiently and sometimes permanently depressed (Ditunno et al., 2004).

Given this latter concern, the current study was designed to assess the contribution of the EPR to the cardiovascular response to exercise immediately after induction of an acute Brown-Séquard-like lesion. The study utilized an animal model (the cat) in which a lateral spinal hemi-section could be readily induced and easily verified. In addition, the model allowed for the activation of the EPR-independent of central command. It was hypothesized that the cardiovascular response to activation of the EPR would be attenuated, to some extent, post induction of SCI. That being stated, it was further postulated that despite reductions in reflex function, the EPR would maintain the ability to significantly contribute to cardiovascular regulation during exercise.

## Materials and methods

Experiments were performed on seven mongrel cats of either sex weighing between 3 and 5 kg. The procedures outlined were approved by the Institutional Animal Care and Use Committee of the University of Texas Southwestern Medical Center at Dallas. All studies were conducted in accordance with the United States Department of Health and Human Services National Institutes of Health Guide for the Care and Use of Laboratory Animals.

### General surgical procedures

Animals were initially anesthetized with isoflurane gas (2–5% in 100% oxygen). Cats were subsequently intubated and artificially ventilated with a mechanical respirator (model 661, Harvard Apparatus). Levels of inhalant gas were increased as indicated by a withdrawal reflex to pinching of the hind paw, presence of a corneal reflex, and/or spontaneous increases in heart rate (HR). Fluid-filled catheters were placed within an external jugular vein for the administration of fluids and a common carotid artery for the measurement of arterial blood pressure (ABP). Arterial blood gas levels were periodically monitored using an automated blood gas analyzer (model ABL5, Radiometer) and were maintained within the following: arterial PO_2_, >80 mmHg; arterial PCO_2_, 35–40 mmHg; pH, 7.3–7.4. Body temperature, as assessed by rectal thermometer, was kept within 37–38°C by a water-perfused heating pad and an external heat lamp. A urinary catheter was inserted to monitor urine output throughout the experiment. Following cannulation, a laminectomy was performed in order to isolate the ventral roots from L_7_–S_1_. Briefly, the dorsal aspect of the L_7_–S_1_ vertebrae were removed and the dorsal and ventral roots were carefully separated. The ventral roots were transected along the peripheral ends and positioned on a bipolar stimulating electrode. A small pouch was formed from the skin of the exposed area and the neural tissue submersed in mineral oil. The triceps surae muscles were isolated in both legs. The calcaneal bone of each leg was sectioned and the Achilles tendons connected to force transducers (Grass FT10). Cats were then placed in a stereotaxic frame (Kopf Instruments) with hip spikes positioned to secure the animal within the frame. A mid-collicular decerebration was performed and anesthesia was withdrawn. At the conclusion of each experiment, animals were humanely euthanized by administration of sodium pentobarbital (120 mg/kg iv).

### EPR testing

The triceps surae muscles of either the right or left hindlimb were contracted for 30 s with a 20 min recovery period between each contraction. Each hindlimb was contracted a minimum of two times. Contractions were produced via electrical stimulation of the L_7_ and S_1_ ventral roots at a frequency of 30–40 Hz at 3 × motor threshold with a pulse duration of 0.1 ms. This procedure has been shown to selectively stimulate both the mechanically and chemically sensitive components of the EPR-independent of central command activation (Mitchell et al., 1983). Following, a lateral hemi-section of the spinal cord was made in the T_13_ to L_2_ region with fine forceps. After a stabilization period of 1 h, the contraction protocol was repeated in each limb. Finally, the cardiovascular responses observed post SCI were confirmed as neural in nature by repeating the contraction protocol in both limbs after severing the dorsal and remaining ventral roots caudal to L_4_.

### Histology

At the conclusion of each experiment, sections of the injured spinal cord were harvested and fixed for histological examination. The spinal cord was sectioned transversely. Each section was 50 μm in thickness. Sections were mounted on slides and stained using Luxol blue to evaluate white matter. These procedures were performed to verify the extent of the experimentally-induced lesion in each animal.

### Data acquisition and statistical analyses

ABP was continuously monitored via a pressure transducer (model P23 ID, Statham). Mean arterial pressure (MAP) was obtained by integrating the arterial signal with a time constant of 4 s. HR was derived from the ABP pulse using a biotachometer (Gould Instruments). A force transducer (model FT10, Grass Instruments) was attached to the Achilles tendon, and used to quantify contraction-induced tension development. All data were subject to analog-to-digital conversion (micro 1401, Cambridge Electronic Design) using commercially available software (Spike 2 version 3, CED) and recorded on a personal computer (550-MHz Pentium III, Dell Computer). All statistical tests were performed using SigmaStat 2.03 for Windows (SPSS). Comparisons between experimental procedures were analyzed using repeated measures analysis of variance (ANOVA) with a Student-Newman-Keuls *post hoc* test employed when the ANOVA was found to be significant. Results are presented as means ± SE. The level of significance was set at *P* < 0.05.

## Results

**Figure 1:**
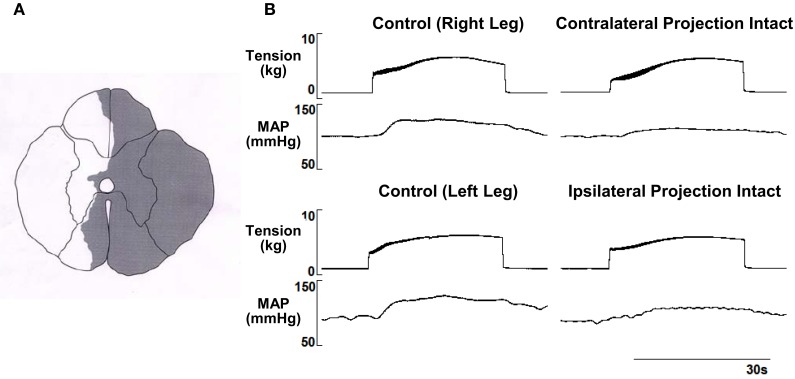
**(A)** Histological reconstruction of the Brown-Séquard like lesion in the lower spinal cord of one animal. **(B)** Electrically-induced static contraction increased MAP before (left tracing) and after (right tracing) induction of the Brown-Séquard like lesion in both the leg ipsilateral to the injury site (contralateral sensory projection intact) and contralateral to the injury site (ipsilateral sensory projection intact).

**Figure 2:**
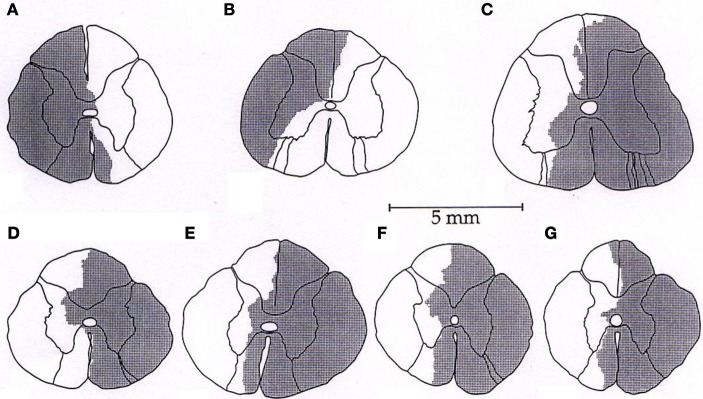
**(A–G)** Histological reconstructions of the Brown-Séquard like lesion in the lower spinal cord of each animal used in the investigation.

**Figure 3:**
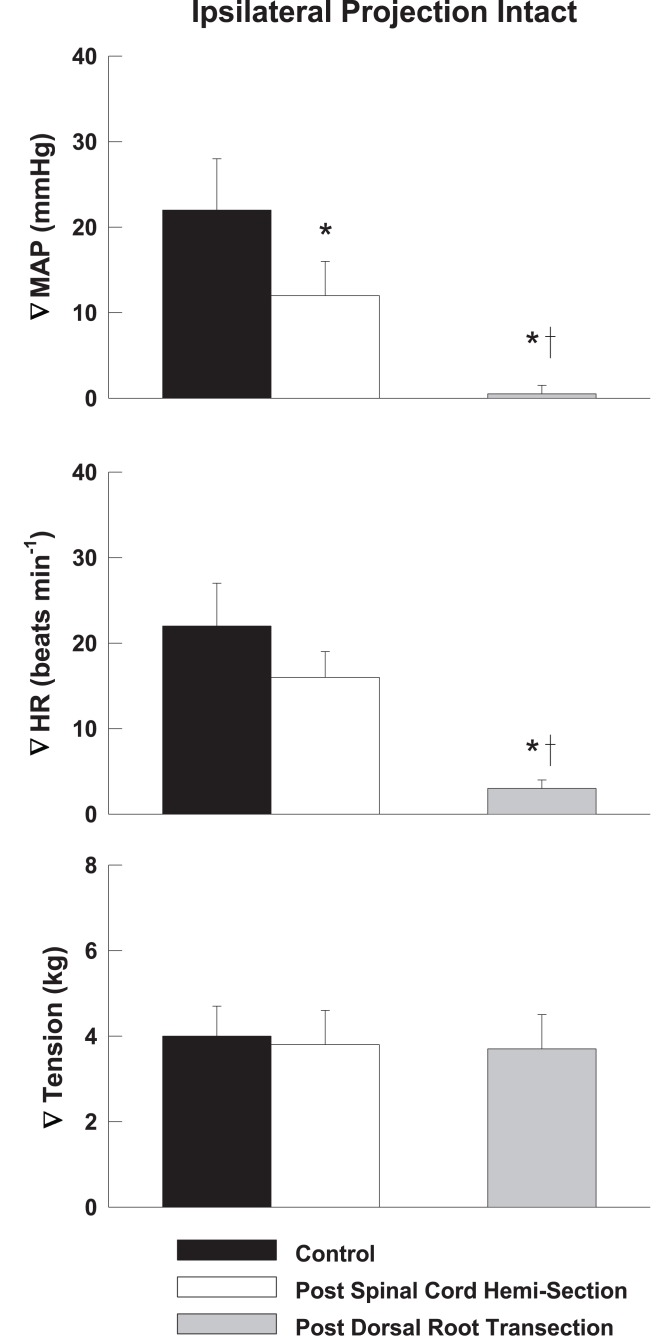
**After lateral hemi-section of the spinal cord, the exercise-induced increases in MAP were attenuated compared to control when contracting the leg contralateral to the lesion (ipsilateral sensory projection intact).** HR responses were largely unaffected. Sectioning the dorsal roots innervating the activated hindlimb skeletal muscle almost completely abolished the cardiovascular response to exercise. Asterisk indicates significantly different from control condition. Cross indicates significantly different from spinal cord hemi-section condition (*P* < 0.05).

**Figure 4:**
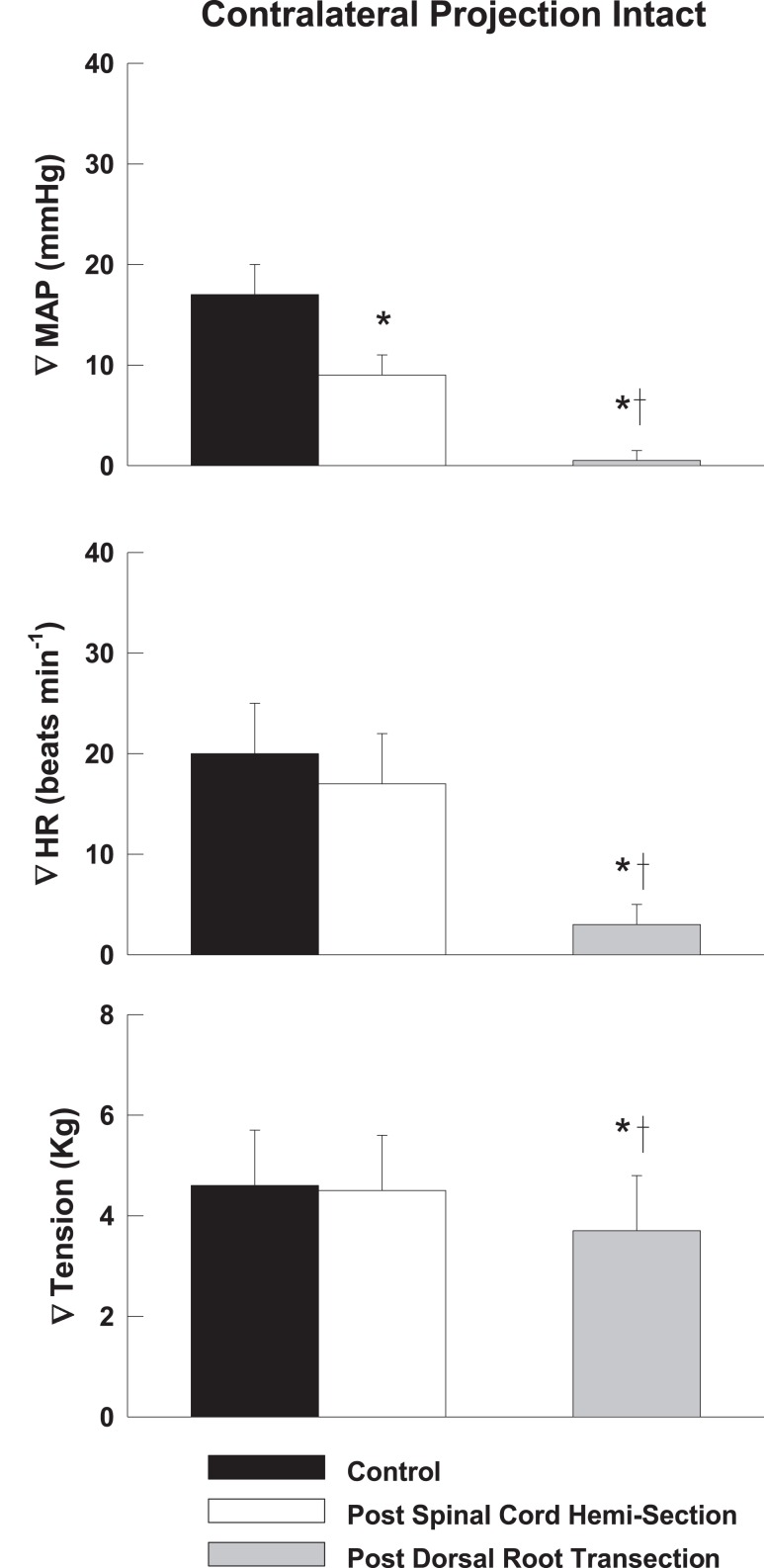
**After lateral hemi-section of the spinal cord, the exercise-induced increases in MAP were attenuated compared to control when contracting the leg ipsilateral to the lesion (contralateral sensory projection intact).** HR responses were largely unaffected. Sectioning the dorsal roots innervating the activated hindlimb skeletal muscle almost completely abolished the cardiovascular response to exercise. Asterisk indicates significantly different from control condition. Cross indicates significantly different from spinal cord hemi-section condition (*P* < 0.05).

Representative tracings from one cat of the MAP responses to electrically-induced static muscle contraction before and after spinal cord hemi-section are presented in Figure 1. Histological confirmation of the extent of the SCI for each animal studied is presented in Figure 2. Baseline blood pressures prior to muscle contraction were not different before or after hemi-section of the spinal cord whereas the induction of SCI significantly raised baseline HR (Table 1). In all conditions, muscle contraction-induced significant increases in MAP, HR, and muscle tension from baseline values (Table 1). The pressor response (12 ± 4 mmHg) to activation of the EPR during contraction of the limb contralateral to the lesion (ipsilateral sensory projection intact) was significantly attenuated compared to the change in pressure evoked by contraction prior to the induction of SCI (23 ± 5 mmHg). Similarly, the HR response to contraction after lateral hemi-section tended to be reduced although statistical significance was not obtained (Figure 3). When contracting the limb ipsilateral to the lesion (contralateral sensory projection intact), the MAP response prior to spinal cord hemi-section (16 ± 3 mmHg) was significantly greater than the change in pressure observed after surgical lesioning (9 ± 2 mmHg). Again, there was a tendency for the HR response to activation of the EPR to be less after induction of injury although a statistical difference was not observed (Figure 4). Interestingly, the magnitude of the reduction in the pressor response to activation of the EPR post-spinal cord hemi-section was similar regardless of the limb contracted in relation to the location of the spinal cord lesion. For example, the MAP response to static contraction was diminished by a little over 40% when contracting the limb either ipsilateral or contralateral to the lesion. In other words, over 50% of the pressor response evoked by muscle contraction prior to injury remained after the induction of injury. Regardless of the limb contracted in relation to the spinal cord lesion, bi-lateral dorsal root transection almost completely abolished the pressor and cardioaccelerator responses to static contraction (Figures 3 and 4).

**Table 1:** **Cardiovascular responses to contraction before and after hemi-section of the spinal cord**.

**Variable**	**Condition**	**Contralateral projection intact**		**Ipsilateral projection intact**	
		**Control**	**Post hemi-section**	**Control**	**Post hemi-section**
MAP	Baseline	140 ± 8	136 ± 9	137 ± 10	133 ± 8
(mmHg)	Peak	156 ± 11^*^	144 ± 9^*^^†^	160 ± 14^*^	145 ± 11^*^^†^
HR	Baseline	198 ± 22	228 ± 16^†^	196 ± 23	234 ± 15^†^
(beats·min^−1^)	Peak	217 ± 22^*^	245 ± 17^*^^†^	217 ± 22^*^	250 ± 14^*^^†^
Tension	Baseline	0.5 ± 0.1	0.5 ± 0.1	0.5 ± 0.1	0.5 ± 0.1
(kg)	Peak	5.1 ± 1.0^*^	5.0 ± 1.0^*^	4.5 ± 0.7^*^	4.3 ± 0.7^*^

Data are means ± S.E.M. (n = 7). MAP, mean arterial pressure; HR, heart rate.

* Significantly different from baseline.

† Significantly different from control. P < 0.05.

## Discussion

This study demonstrates, in cats, that the EPR significantly contributes to the pressor and cardioaccelerator responses to muscle contraction immediately following lateral spinal cord hemi-section independent of central command. The MAP responses to either electrically-induced static contraction of the limb contralateral to the lesion (ipsilateral sensory projection intact) or ipsilateral to the lesion (contralateral sensory projection intact) were reduced after SCI. That being stated, a significant proportion of the pressor response (over 50%) remained post hemi-section regardless of the limb contracted in relation to the site of the lesion. Interestingly, with regard to HR, the induction of acute SCI had no significant effect on the tachycardic response to activation of the EPR. These findings suggest that the EPR largely maintains the ability to elicit the pressor and tachycardic responses required to support exercise directly after a lateral injury to the spinal cord. The results further suggest that depression of the EPR by acute spinal shock is minimal if present at all.

Previously, Winchester et al. (2000) recruited four patients with Brown-Séquard syndrome (i.e., lateral spinal cord hemi-section) to assess the contributions of central command and the EPR to the cardiovascular response to static exercise. Due to the fact that these patients presented with a loss of sensation and intact motor control on one side of the body and intact sensation and a loss of motor control on the other side of the body, these investigators were able to perform a series of contractions under four unique conditions: (1) voluntary contraction of the sensory loss/motor intact leg (central command activated, EPR input minimal); (2) electrical stimulation of the sensory loss/motor intact leg (no central command, EPR input minimal); (3) attempted voluntary contraction of the sensory intact/motor loss leg (central command and EPR activated); and (4) electrical stimulation of sensory intact/motor loss leg (no central command, EPR activated). Isometric knee extension exercise-induced increases in ABP and HR, albeit reduced in magnitude compared to that reported in the literature for normal healthy individuals, under each of these conditions with the exception of electrical stimulation of the sensory loss/motor intact leg. In their interpretation of these findings, the investigators suggested that both central command and the EPR maintained, to some extent, their ability to regulate the cardiovascular system during exercise in a chronic SCI patient population. With regard to the EPR, the results of the current investigation suggest that the findings of Winchester and colleagues may be extended to the acute phase of lateral SCI.

In agreement with a previous report based on a small number of animals (Iwamoto et al., 1984), the findings of the current investigation suggest that, in cats, the spinal pathway mediating the EPR control of MAP is bilateral in nature implying a level of redundancy within this species. The extent of this pathway redundancy appears to be incomplete with regard to blood pressure as the absence of either of its components (i.e., contralateral or ipsilateral sensory projection) attenuates the pressor response to static muscle contraction. That being stated, the data also suggest that neither component of the bilateral pathway is dominant as interrupting either the ipsilateral or contralateral sensory projection in relation to the exercising limb produces the same decrement in the MAP response to contraction. These conclusions do not appear to be applicable to the tachycardic response to EPR activation as it was predominately unaffected by spinal cord lesioning.

Unlike the cat, the EPR pathway in humans appears to be predominately unilateral in nature (Winchester et al., 2000). This must be considered when interpreting and translating the findings of the current investigation to humans suffering an acute lateral hemi-section of the spinal cord. That being stated, the known characteristics of spinal cord pathways may aid in relating the functional, physiological findings in cats to humans with a Brown-Séquard lesion. Three pathways are of principal interest: the spinothalamic, spinoreticular, and spinomesencephalic (including that to the periaqueductal gray) tracts. Each of these pathways is generally characterized (with some variation depending on target nucleus) by a strong component which ascends contralaterally from spinal levels in the primate and cat with a lesser ipsilateral projection (Willis and Coggeshall, 2004). At least in the case of the spinothalamic tract, it is clear that the ipsilateral projection in the primate (a close relative of humans) is proportionally smaller than in the cat (Willis and Coggeshall, 2004). This may account, in part, for the lack of a cardiovascular response evoked during electrically-induced exercise in the limb contralateral to the lesion in the Brown-Séquard patient study previously discussed (Winchester et al., 2000). It is somewhat more difficult to make this association with the other tracts, but the differences between the cat and the primate pathways (and hence possibly humans) appear to be similar to that noted in the spinothalamic tract (Willis and Coggeshall, 2004). However, it is evident from the current study that ipsilateral projections in the cat are capable of mediating a strong EPR-mediated cardiovascular response. As mentioned, in the human Brown-Séquard investigation, patients were studied months to years after their initial lesion in direct contrast to the acute injuries-induced in the present experiments (Winchester et al., 2000). Although contralateral projections predominate in the spinal tracts of primates, lesser ipsilateral projections exist that may also be present in humans (Willis and Coggeshall, 2004). It is unknown whether activation of such ipsilateral pathways by the EPR could elicit cardiovascular responses after acute injury in patients as has been demonstrated in the cats of the current study. Likewise, it is unknown what effect beginning exercise training shortly after the occurrence of SCI might have on surviving ipsilaterally projecting cells in humans. Although highly speculative, it is possible that enhancing the activity of ipsilateral projections in the EPR pathway shortly after injury in humans could induce a functional plasticity that effectively compensates for the loss of the more dominant contralateral projections. Similar plasticity has been demonstrated in efferent projections to the phrenic motor nucleus in which respiratory challenge facilitates activity in contralateral neurons that restores function to the phrenic motor neurons on the side ipsilateral to the lesion (Fuller et al., 2006). As a result, diaphragmatic activity is recovered. Increasing our understanding of how the cat utilizes these ipsilateral EPR pathways after acute spinal cord hemi-section may facilitate our ability to induce similar plasticity in humans shortly after injury.

It should likewise be noted that the findings of the current investigation in cats and those previously reported in Brown-Séquard patients (Winchester et al., 2000) are primarily confined in application to individuals experiencing a complete lateral hemi-section of the spinal cord. Circumstances in which the spinal cord is only partially lesioned in any portion or completely transected would most likely produce different results. On the other hand, it is possible that EPR-mediated cardiovascular responses might not be different under these specific SCI conditions if extraspinal pathways (perhaps carried in the sympathetic trunk) are utilized. Studies in which EPR function is assessed after complete transection of the spinal cord could address this possibility. In addition, the level of the spinal cord in which the injury is sustained may also affect the function of the EPR. That being stated, the Brown-Séquard patients studied previously displayed similar cardiovascular responses to exercise under various conditions with hemi-sections ranging from the C5 to T8 neurological levels.

Limitations that could affect the interpretation of results in this study are acknowledged. To begin, the contribution of central command to the cardiovascular response to exercise after acute spinal cord hemi-section was not examined in this investigation. The importance of this input to circulatory regulation during physical activity immediately following SCI warrants further investigation. Secondly, due to technical reasons, arterial PCO_2_ was maintained within the range of 35–40 mmHg. This is slightly elevated from the PCO_2_ values reported in normal conscious cats which tend to be in the lower 30 mmHg range (Herbert and Mitchell, 1971; Lovering et al., 2003). As a result, sensory input from central and peripheral chemoreceptors that effect both respiratory and cardiovascular function could have influenced the hemodynamic responses to muscle contraction reported. That being stated, as arterial PCO_2_ was elevated only minimally, this concern is relatively minor. Thirdly, both baseline HR and MAP tended to be on the high end of the range reported commonly for decerebrated cats (Iwamoto et al., 1985; Hayes and Kaufman, 2001; Hanna and Kaufman, 2003). It is possible that such elevated basal hemodynamics may have limited the magnitude of the EPR-induced pressor and tachycardic responses masking the full impact of the spinal cord hemi-section. For example, changes in MAP are commonly reported to be in the range of 30–40 mmHg in response to muscle contraction in decerebrated cats (Iwamoto et al., 1985; Hayes and Kaufman, 2001; Hanna and Kaufman, 2003). The group means for the MAP responses to EPR activation prior to hemi-section in the current study were approximately 15–25 mmHg. In contrast, the HR responses elicited were similar to those previously reported (Iwamoto et al., 1985; Hayes and Kaufman, 2001). Finally, this study was conducted without the inclusion of a time control (i.e., conduction of the protocol over the full time period without the induction of spinal hemi-section). That being stated, previous studies in decerebrate cats have reported no decrement in the pressor or tachycardic response to EPR activation for several hours after initial stimulation (Hayes and Kaufman, 2001). Similar results have been reported in anesthetized cats (Smith et al., 2005).

### Clinical implications

As stated previously, injuries to the spinal cord, whether complete or incomplete, often disrupt cardiovascular control and induce a host of complications. The autonomic dysfunction that develops can be associated with a number of conditions that increase the risk for cardiovascular disease including abnormalities in blood pressure related to central and peripheral vascular dysfunction, irregularities in HR variability, generation of arrhythmias and abnormal temperature control (Gao et al., 2002; Jacobs and Nash, 2004; Myers et al., 2007; Brown and Macefield, 2008; Alexander et al., 2009). In addition, the cardiovascular response to exercise is often blunted resulting in decreased exercise capacity which can subsequently increase inactivity (Myers et al., 2007). Physical inactivity in itself can lead to an elevated risk for thromboembolism, due to the development of coagulation disorders and venous stasis, as well as obesity and metabolic syndromes (Waring and Karunas, 1991; Groah et al., 2001; Myers et al., 2007; Ploumis et al., 2009). Prevention and/or successful treatment of these cardiovascular complications following SCI could clearly reduce the incidence of cardiovascular disease in this patient population. To this end, evidence supports the use of exercise as an effective therapy for enhancing physical capacity, muscular strength, and vascular function in the SCI patient (Gerrits et al., 2001; Hopman et al., 2002; DeGroot et al., 2005; Thijssen et al., 2005, 2006; Zbogar et al., 2008; Hicks et al., 2011; Phillips et al., 2011). It is logical to propose that the earlier the exercise therapy begins, the more effective the treatment. The results of the current investigation suggest that regulation of the cardiovascular system by the EPR is relatively maintained immediately after acute lateral hemi-section of the spinal cord and can likely support the metabolic demands of physical activity. The findings increase our understanding of both the mechanisms of neural cardiovascular control during exercise post SCI as well as the time at which this control is operative. The results may prove beneficial in the development of training interventions specifically designed to optimize the efficacy of physical activity as a therapeutic modality in these patients.

### Summary

In cats with an acute lateral hemi-section of the spinal cord, activation of the EPR by electrically-induced static exercise increases MAP and HR. The responses can be evoked by contracting skeletal muscle in either the limb contralateral to the lesion (ipsilateral sensory projection intact) or ipsilateral to the lesion (contralateral sensory projection intact). These findings suggest that the EPR maintains the potential to contribute significantly and importantly to the cardiovascular response to exercise immediately following spinal cord hemi-section.

### Conflict of interest statement

The authors declare that the research was conducted in the absence of any commercial or financial relationships that could be construed as a potential conflict of interest.
